# Multifactorial approaches to study bilingualism in the aging population: Past, present, future

**DOI:** 10.3389/fpsyg.2022.917959

**Published:** 2022-07-29

**Authors:** Tanya Dash, Yves Joanette, Ana Inés Ansaldo

**Affiliations:** ^1^Centre de recherche de l'Institut Universitaire de Gériatrie de Montréal, Montreal, QC, Canada; ^2^École d'orthophonie et d'audiologie, Faculté de médecine, Université de Montréal, Montreal, QC, Canada

**Keywords:** multifactorial approach, subjective measures of bilingualism, objective measures of bilingualism, cognitive performance, confounding variables

## Abstract

A better understanding and more reliable classification of bilinguals has been progressively achieved through the fine-tuning methodology and simultaneously optimizing the measurement tools. However, the current understanding is far from generalization to a larger population varying in different measures of bilingualism—L2 Age of acquisition (L2 AOA), L2 usage and exposure, and L2 proficiency. More recent studies have highlighted the importance of modeling bilingualism as a continuous variable. An in-depth look at the role of bilingualism, comparing groups, may be considered a reductionist approach, i.e., grouping based on one measure of bilingualism (e.g., L2 AOA) may not account for variability in other measures of bilingualism (L2 exposure, L2 use or L2 proficiency, amongst others) within and between groups. Similarly, a multifactorial dimension is associated with cognitive performance, where not all domains of cognition and subcomponents are equally influenced by bilingualism. In addition, socio-cultural and demographical factors may add another dimension to the impact of bilingualism on cognitive performance, especially in older adults. Nevertheless, not many studies have controlled or used the multiple socio-cultural and demographical factors as a covariate to understand the role of different aspects of bilingualism that may influence cognitive performance differently. Such an approach would fail to generalize the research findings to a larger group of bilinguals. In the present review paper, we illustrate that considering a multifactorial approach to different dimensions of bilingual study may lead to a better understanding of the role of bilingualism on cognitive performance. With the evolution of various fine-tuned methodological approaches, there is a greater need to study variability in bilingual profiles that can help generalize the result universally.

## Introduction

Over the years, studies have demonstrated that bilingualism improves cognitive performance, particularly in older persons (Bialystok, 2021a). However, the role of bilingualism in enhanced cognitive performance has been highly debated. On the one hand, researchers have provided empirical evidence demonstrating faster and more accurate performance in bilingual participants compared to their monolingual peers on a variety of cognitive tasks (Pliatsikas and Luk, 2016; Dash et al., 2019). On the other hand, opponents have failed to replicate the expected group differences (Paap et al., 2018), implying spurious findings. Furthermore, various meta-analyses assessing the link between bilingualism and cognitive performance have supported the accuracy of positive results (Adesope et al., 2010; Baumgart and Billick, 2018; Grundy, 2020) and the null results (Paap and Greenberg, 2013; van den Noort et al., 2019), adding to the unresolved controversy. Most null results come from the behavioral data testing younger bilinguals. However, these research results (both positive and null) need to be interpreted cautiously while acknowledging the individual differences that may exist within and across groups when participants are classified based on a simple binary question. The discrepancies in literature lie in methodological and conceptual understanding, among others, lack of second language competency information and conflicting classification criteria (Grosjean, 1998; Grundy, 2020). In addition, measures of cognitive performance used in bilingual literature vary across studies. These cognitive tasks usually assess different subcomponents of attention, cognitive control, or working memory and are also assessed in verbal or non-verbal modalities adding to the complexity of interaction between language and cognition (Dash et al., 2022). Moreover, there is a lack of standard practices to identify and control confounding socio-cultural and demographic variables in exploring the consequences and antecedents of bilingualism. The present review, thus, sought to assess the evolution of methodological rigor and conceptual understanding of bilingualism and its relation to cognitive performance with a focus on the aging population. Under various subheadings, this review will focus on two main aspects: (1) illustration of different strategies to profile the bilingual population and (2) to demonstrate different indices of the cognitive performance in bilinguals. In doing so, this review highlights the limitation of existing approaches and the use of a multifactorial approach to measuring levels of bilingualism and the related cognitive ability in different domains of cognition. The multifactorial approach to studying bilingualism in the aging population finds support in the concept of *emergentism* described by Hernandez and colleagues in reference to bilingualism (Hernandez et al., 2018, 2019; Claussenius-Kalman et al., 2021). *Emergentism* is a philosophical concept originally described by Mill (1843) in a physical system, where dynamic forces combine to form simple motion. *Emergentism* can also be used to explain the learning of second languages (Gregg, 2004; MacWhinney, 2002). More broadly speaking, *emergentism* in the context of bilingualism can be described as an interaction between the ecosystem and expertise of the learner during second language acquisition. *Emergentism* refers to an interaction between the ecosystem and expertise of the learner during second language acquisition. The term “ecosystem” refers to the characteristics of the second language learning environment, i.e., language usage, frequency, similarities between languages, and mode/environment of learning. On the other hand, “expertise” refers to the learner's aptitude for learning a new language. This includes age and individual differences in cognitive skills like memory, cognitive control, and cognitive flexibility. Emergentism takes a developmental perspective indicating an interaction between *ecosystem* and *expertise* that results in a variable outcome of bilingual language processing. Thus, each second language learner has a different developmental trajectory represented in a unique multidimensional space, depending on the interaction between their ecosystem and ability. Thus, a multifactorial approach allows the researchers to account for the inter-individual variability in the bilingual population by adding an assessment of multiple factors related to bilingual experiences, demographic strata, and cognitive performance simultaneously. Although, we have seen an evolution in the approach to quantifying bilingualism using different tools and methods (Dash et al., 2019, 2022; Gullifer and Titone, 2020; Sulpizio et al., 2020; Macdonald et al., 2022), with restrictions on the use of a single test to assess cognitive performance with only a few studies trying to control for confounding variables.

## Toward the understanding of bilingual phenotype

Over the years, evolutionary changes have occurred in the theoretical and methodological ways to characterize bilingualism. Defining bilingualism becomes more complicated when considering what “*knowing a language*” means and how one can define various aspects of bilingualism. When defining bilingualism, researchers often rely on multiple measures of bilingualism, such as the L2 AOA, L2 language usage and exposure, and L2 proficiency (Marian et al., 2007; Li et al., 2014; Anderson et al., 2018; Dash et al., 2019, 2022; Marian and Hayakawa, 2021). Different measures of bilingualism are often interrelated, and given the heterogeneity in the bilingual experience, interrelation may not follow the same trajectory. Therefore, a multidimensional and dynamic phenomenon of bilingualism needs a holistic multifactorial approach to capture the inherent nature of the bilingual experience, more so in the aging population, as the accuracy of reporting bilingual experience may introduce additional variability. In the past several decades, bilingual literature has evolved from a dichotomous to a continuum approach to defining and modeling bilingualism. Researchers have found that the heterogeneity in the traditional approach to categorizing participants in a bilingual and monolingual group may result in inconsistent findings in cognitive performance between the groups (Baum and Titone, 2014; Luk, 2015; de Bruin, 2019; DeLuca et al., 2019). In addition, many studies have failed to find the benefits of bilingualism on cognitive performance in the aging population (Olsen et al., 2015; Keijzer and Schmid, 2016; Papageorgiou et al., 2019; Soltani et al., 2021). The reason for the inconsistency may originate in the way groups are labeled and thus classified. Surrain and Luk (2019) highlights different ways in which researchers have classified their bilingual group; it was evident that 77% of the studies use the label “*bilingual*” or “*specific language pair bilingual*,” and only minimal studies (19%) use combination of factors to label the bilingual group. Another evolutionary transition was understanding variations in cognitive performance within bilingual groups. Various behavioral and neuroimaging studies have compared two extremes of the population within the bilingual category—high vs. low proficiency (Singh and Mishra, 2013), balanced vs. unbalanced (Woumans et al., 2015), early vs. late (Tao et al., 2011). Although such an approach still categorizes the participants into two groups, it has led to much informative literature on bilingualism. The debatable role of bilingualism in cognitive performance also stems from the variability in bilingual experiences; for example, a high proficient bilingual may be an early or late bilingual, or an early bilingual may be an unbalanced bilingual. Therefore, the prediction made using one set of observable variables (for example, proficiency) does not apply to another set of observable variables (for example, language usage), thus limiting reliable and replicable research findings.

Recent studies have used statistical methods to mathematically combine and use continuous variables to predict changes in cognitive performance (Gullifer et al., 2018; Dash et al., 2019, 2022). Moreover, the use of statistical methods to determine outcomes for the measure of bilingualism has found support in a recent study by Macdonald et al. (2022). Authors find convergence between outcomes from various statistical methods (like confirmatory factor analyses and latent profile analyses) and another continuous metric of bilingualism (Vaughn and Hernandez, 2018) and self-reported information (Macdonald et al., 2022). Since bilingualism is a multidimensional construct, there can be an overlapping continuum of different measures of bilingualism. Similarly, a bilingual continuum created using one dimension of bilingual experience (for example, language usage, DeLuca et al., 2019) may have a different trajectory in another dimension. Furthermore, the lack of consensus in bilingual literature also stems from the differences in how different measurement tools are used to study bilingualism. Therefore, it is crucial to determine which task and stimuli are used as measures of bilingualism, and once scholars determine the variables of interest, the next logical step is to figure out how they use them to understand the role of bilingualism in cognitive performance. Depending on the research questions, researchers have often used 1 or 2 measures to categorize participants into different groups; more recent studies use different bilingualism measures on a continuum. Categorizing participants in groups allows for simplification of the analyses, presentation, and interpretation of the results from a study (DeCoster et al., 2011). The data presentation is easier by dichotomizing the variables using a table or graph with the mean scores to demonstrate differences between groups. However, If the predictor variable is continuous, then the slope of the predictor variable with the outcome variable needs to be presented using regression lines. For example, to explore the interaction effect between age and bilingualism on cognitive performance, a researcher may construct distinct regression lines between bilingualism and cognitive performance for different age cohorts (young vs. older adults) and interpret the effect. In addition, when age and bilingualism vary continuously, the statistical approach to presentation needs to be tweaked. Such methods are more complicated than presenting group means. Similarly, categorical analysis is typically more straightforward and traditional than continuous analysis. ANOVA, which requires a categorical predictor variable, is more commonly used by psychologists to test influences on an outcome variable. However, the linear mixed effect model (Gallo et al., 2022) and growth curve analysis (Incera and McLennan, 2017) are gaining popularity in recent times where multiple continuous variables can also be considered to predict the outcome. Some potential arguments in favor of categorization were provided by Farrington and Loeber (2000). They propose that arbitrarily categorizing variables is one method for dealing with variables with highly skewed distributions or when the relationship between predictor and outcome variable is not linear. However, there are more cons than pros in using measures of bilingualism to categorize participants. To begin with, conducting group analyses when the variable of interest may vary on a continuum diminishes statistical power and increases the risk of rejecting the null hypothesis (Cohen, 1983; Altman and Royston, 2006; DeCoster et al., 2011). Secondly, universally accepted grouping criteria are unavailable, limiting the reproducibility of the results in different studies (Altman and Royston, 2006). Especially with the aging population, categorizing participants based on the current language usage and proficiency may ignore the necessary bilingual experience (spanning over decades) crucial for building an accurate bilingual profile. Furthermore, suppose the split is made at an arbitrary cut-off point (say, the median age of acquisition of 10 years). In that case, participants with an age of acquisition of 9 and 11 years are placed in different groups, even though they may be more like each other than other members of their group (i.e., age of acquisition of 9 years is more similar to that of 11 years than that of 1 year; MacCallum et al., 2002; Altman and Royston, 2006).

To summarize, grouping bilinguals when the underlying construct is continuous has statistical implications and may obfuscate our understanding of the measure of bilingualism in the research study. It is comparatively easy to group participants; however, it adds researchers' bias to the study. Finally, when groups are constructed based on the values of a continuous measure, a significant amount of information and variability that may exist within a group are lost (MacCallum et al., 2002). In the following sections, we will elaborate on the most commonly used tools available to measure bilingualism and how these tools are used to classify participants or create a continuum.

## Measures of bilingualism: Subjective and objective measures

While highlighting the lack of consensus between the research fraternity on the role of bilingualism in cognition, this section will enumerate different tools used to measure bilingualism. The selection of different measurement variables is based on the way researchers have defined bilingualism and the measures used to ascertain the inclusion of participants. There are currently multiple ways to measure bilingual experience (de Bruin, 2019), broadly classified into subjective and objective measures. Self-report measures of bilingualism are the most widely used tools in various studies across different bilingual populations (Grundy, 2020; Kremin and Byers-Heinlein, 2021). The most used questionnaires for adult bilinguals are Language History Questionnaire (LHQ; Li et al., 2006, 2014, 2020), Language Experience and Proficiency Questionnaire (LEAP-Q; Marian et al., 2007), and Language and Social Background Questionnaire (Luk and Bialystok, 2013; Anderson et al., 2018). Although not identical, these questionnaires assess fundamental measures of bilingual experience—L2 AOA, language usage and proficiency, and language immersion. Some of the key differences between these questionnaires are how responses are recorded and the range of the rating scale (varies from 5 to 10 Likert scale; use of descriptive terms like “more,” strongly agree). LEAP-Q is explicitly designed to measure speech and language skills; hence it also assesses self-perception of accent in participants' speech which is missing in other questionnaires. LEAP-Q provides an extensive set of questions that different research groups can use differently. LHQ, on the other hand, reports language background, proficiency, usage, and dominance; and provides an aggregate score for proficiency, dominance, and immersion. LSBQ is specifically designed for countries with an immigrant population, and the questions focus on the extent of non-English language proficiency and use at home and in other social situations. LSBQ and LHQ have developed a revised version focusing on the interpretation guide and recommended cut-off scores for the continuous outcome variable into categorical groups.

L2 AOA is the commonly used variable to categorize participants into respective groups (bilingual vs. monolingual; early vs. late bilinguals) to compare the executive functioning in the aging population (Luk et al., 2011; Bak et al., 2014; Ansaldo et al., 2015). L2 AOA is a static variable that is comparatively easier to report than L2 proficiency and usage, which may experience dynamic change throughout the language learning experience, especially in the aging population. While reporting L2 AOA, some participants may estimate L2 AoA based on early exposure to the second language (e.g., parents, friends, music, and television); others may indicate the start of formal classroom learning. Furthermore, studies frequently use the age of immigration to a new country (Tao et al., 2011) as an indicator of L2 AOA. Interestingly, previous studies from 2005 to 2015 have more frequently reported L2 proficiency and usage variables (77 and 79%) than L2 AOA (67%; Surrain and Luk, 2019). Most questionnaires measure second language usage or exposure in general and interactional contexts (e.g., language use at home, work, social setting). Researchers have predominantly used raw scores (percentage exposure, rating on the Likert scale) or normalized scores as an indicator of language usage to predict cognitive performance. Estimating the frequency with which each language is used daily is difficult, but it is even more complicated when bilinguals vary the use of a particular language depending on the context of language usage (Grosjean, 1998; Green and Abutalebi, 2013). To obtain a more comprehensive language usage scores, questionnaires often assess exposure and usage in diverse situations, such as with different interlocutors (e.g., family, friends), at different stages of life (e.g., primary school, high school), and topics (e.g., emotions, leisure activities, media). In addition, studies have categorized bilingual participants under three interaction contexts: Single-language contexts, dual-language contexts, and dense code-switching contexts based on the interaction of language usage frequencies in various contexts (Green and Abutalebi, 2013; Lai and O'Brien, 2020). Rodriguez-Fornells et al. (2012) developed the Bilingual Switching Questionnaire (BSWQ) to assess better language context, which examines multiple aspects of code-switching. The BSWQ helps categorize participants into four categories: L1 switcher, L2 switcher, contextual switcher, and accidental switcher. The LSBQ (Anderson et al., 2018) also assesses code-switching and provides composite scores to classify the bilingual population or utilize the measurements on a continuum, along with other measures. Another crucial measure assessed in questionnaires is the L2 language proficiency assessing differences in executive functioning between high and low proficient bilinguals (Singh and Mishra, 2012, 2013). Second language proficiency is usually measured on a Likert scale (for example, 1–7 or 1–10), with an association between self-reported L2 proficiency and standardized language tests is moderate to strong in most questionnaires (Marian et al., 2007; Li et al., 2014, 2020). In comparison, de Bruin et al. (2017) discovered a small to marginally moderate correlation and established objective language assessments (productive vocabulary, receptive vocabulary, and fluency measured in an interview). Given that the participants are estimating their response to the questionnaire after several years, reporting of L2 AoA, usage, and proficiency may encounter over-and under-estimation of self-reported competency, resulting in a lack of association between self-reported measures and conventional language tests (Dash and Kar, 2012; Tomoschuk et al., 2019). Most of these questionnaires do ask specific questions about the language exposure and usage history crucial while understanding bilingual experience in the aging population (i.e., number of years in a second language country, family, school). For example, an elderly, highly proficient bilingual who has gained proficiency over decades of L2 exposure but may not be an active L2 user in the present day and may contribute to a lack of correlation between self-reported information (minimal L2 usage in daily life) and conventional language tests (high scores because of higher language skills). Thus, it is crucial to assess the self-reported variable–L2 usage & proficiency–in greater detail in the aging population. Elderly bilinguals are subjected to intra-individual variability of bilingual experience across different phases of their life that is rarely addressed in research studies.

On the other hand, recent research has recommended using objective measures criteria to assess bilinguals' multifactorial experience (de Bruin, 2019; Tomoschuk et al., 2019; Dash et al., 2022) along with self-reported questionnaires. Picture naming task (Ali et al., 2022), lexical decision time (Pérez et al., 2013), verbal fluency (Suarez et al., 2014), and discourse performances (Dash et al., 2019, 2022) are some of the tasks used to classify participants in different groups or a continuum. MINT (Gollan et al., 2012), the Boston Naming Test (Goodglass et al., 2001), and the Peabody Picture Vocabulary Test (Dunn and Dunn, 1997) are the most common standardized measures of expressive naming ability. These tests are usually available in various languages and have been validated. Another standardized task that is gaining popularity and is available in multiple languages is the LexTALE task (Lemhöfer and Broersma, 2012), which measures receptive vocabulary. It is also vital to recognize that standardized, objective proficiency measurements may limit application in less-studied languages where norms are not easily available. In an attempt to add objectivity to L2 language usage in an interactional context targeting the language switching behavior, researchers have utilized a more ecologically valid technique (EMA, e.g., Shiffman et al., 2008), asking participants to report the frequency of language switching every 2 h for 2 weeks using a smartphone application (Jylkkä et al., 2020). Compared to other questionnaires, assessing switching behavior with an objective tool gives a daily assessment of language switching ability and more accurately captures nuances. Furthermore, numerous objective measures (e.g., production and comprehension, vocabulary, general fluency, etc.) in combination will highlight the multidimensional nature of proficiency. Language proficiency is a multifaceted concept that cannot be reduced to a single metric like naming ability. Multiple objective indices are required to measure language proficiency because a single objective task (usually assessing naming) has a low correlation with the self-reported measure of proficiency (Marian et al., 2007). de Bruin et al. (2017) found that using four objective tasks could better classify bilinguals. Although objective measures of bilingualism are considered important in quantifying bilingual experience, they are rarely reported in bilingual literature. Surrain and Luk (2019) reported that around 38% of the studies assessing language proficiency had provided objective scores. Similarly, Hulstijn (2012) estimated that 45% of studies published in *Bilingualism: Language and Cognition* used objective measures to define language proficiency. However, objective measures of proficiency may benefit from the following recommendations. Firstly, using a single measure of objective language proficiency may not indicate a level of bilingualism. Therefore, it is recommended to use multiple objective measures to create a holistic profile of the bilingual experience (de Bruin, 2019; de Bruin et al., 2021). Secondly, using standardized proficiency measures may not be possible in a different scenario. For example, less popular language combinations may not have standardized tools available in their languages. Also, the use of standardized tools is complicated in studies where multiple language combinations are used. Therefore, we recommend substantiating objective measures with extensive subjective information while assessing bilingual language experience. Simultaneously, there is a greater need to develop tools that apply to different language combinations. For example, the discourse production task as a measure of L2 language proficiency can be considered a holistic measure that can simultaneously provide proficiency scores based on participants' grammatical knowledge, vocabulary skills, organization of content, and fluency. Also, previous studies using L2 discourse proficiency have supported the role of bilingualism in cognitive performances and functional connectivity matrices (Dash et al., 2019, 2022), supporting the use of discourse proficiency as a putative tool. Finally, extensive questionnaires assessing self-reported proficiency information and multiple objective measures tend to increase the number of observable variables in the study. Therefore, it is necessary to substantiate and find appropriate statistical methods to combine the number of observable variables in a meaningful manner. By doing a factorial analysis, these measurements can be merged and utilized to estimate L2 language proficiency levels that can be used as a continuous or categorical measure of bilingualism (Dash et al., 2019, 2022; Calabria et al., 2020). In sum, using a multifactorial approach tapping distinct aspects of bilingualism–L2 AOA, L2 language history, L2 language usage and proficiency, L2 immersion–using multiple self-reported and objective measures may provide a holistic bilingual profile.

## Mathematical and statistical ways to combine bilingual variables

It is widely accepted that individual bilinguals' language experiences are diverse, with unique contexts of acquisition, variations in language usage, and proficiency across the lifespan that can impact socio-cultural identity and cognitive and brain function. The diversity in language experience has led to numerous tools to capture the bilingual experience and has similarly led to corresponding mathematical and statistical ways to use the information collected through different questionnaires and proficiency measures to effectively quantify as a single variable or multiple composite variables for further analysis looking at the impact of bilingualism.

As described above, L2 AOA is one of the commonly used measures subjected to cut-off age to create arbitrary categorization. One common approach is assigning a cut-off to the variable of interest and eventually categorizing the participants or using the cut-off for initial screening. However, using an arbitrary cut-off usually led to discrepancies. For example, an early bilingual label is given to participants with L2 age of acquisition below 5 (Champoux-Larsson and Dylman, 2021), 6 years (Tao et al., 2011; Kalia et al., 2018), 7 years (Pelham and Abrams, 2014), 13 years (Baker and Trofimovich, 2005). Some previous studies had defined early bilinguals when their L2 age of acquisition was prior to the fixed cut-off age and late bilinguals when they acquire their L2 after the cut-off age (Kalia et al., 2018; Champoux-Larsson and Dylman, 2021). The use of a cut-off score is often considered to categorize participants into monolingual and bilingual groups. However, when the language experience of the bilingual and monolingual groups is explored further, heterogeneity within each group may emerge. Bilinguals, for example, may have different ages of acquisition and levels of language usage and proficiency, and monolinguals may have some amounts of exposure to a second language (L2), more so in the aging population where there might be foreign language education in school/college. Given the multifactorial nature of bilingual experience and how different bilingualism measures may interact, it is critical to find ways to synthesize an acceptable number of dependent variables while profiling bilinguals. Thus, traditionally defined bilingual and monolingual groups using arbitrary cut-off points may obscure within-group differences in performance (e.g., MacCallum et al., 2002; Abutalebi and Rietbergen, 2014; Baum and Titone, 2014; Luk, 2015; de Bruin, 2019; DeLuca et al., 2019). Studies have mathematically and statistically combined information to find an appropriate bilingual score.

Many researchers have advocated the need for appropriate guidelines to use questionnaire data and create an independent scoring system. Among different questionnaires used in the literature, the latest version of LHQ (LHQ3.0) provides a user-friendly web-based interface. The only tool available that provides a step-by-step guide for the researchers to calculate an aggregate score to represent participants' overall proficiency, dominance, and immersion levels in each language. These aggregate scores are calculated by normalizing the scores using an appropriate scaling factor (for example, cumulative proficiency score is calculated using a 1-7 Likert scale, so a 1/7 scaling factor is used in the equation), current age, age of acquisition of the language and years and hours of usage of the language. LHQ also provides a ratio score for language dominance in reference to other languages known to the participant. LHQ also allows the researchers to manipulate the weightage of certain variables in the equation based on the research question. For example, if the researcher is interested in bilingual reading and writing proficiency, the aggregate proficiency score is calculated by applying equal weight to reading and writing scores without considering self-reported speaking and understanding. LHQ3.0 has evolved as a one-stop holistic tool that can provide researchers with the flexibility to calculate a single bilingual score suitable for further analysis based on their research question. Another method proposed by Gullifer and Titone (2020) suggests using the *Language Entropy* score as a derived measure of bilingualism based on language usage data in an interactional social setting collected in the questionnaire. Language entropy is measured by calculating proportion scores in different language contexts, i.e., by dividing the L2 rating (for example, 5 on a 1-7 Likert scale) by total rating in different languages (i.e., combining self-reported rating in L1 and L2), followed by calculation of Shannon entropy (H) using the proportion score (see details in language Entropy R package; Gullifer and Titone, 2018). Gullifer and Titone (2020) have argued that language entropy is ideal for synthesizing theoretically relevant variables on a continuum while accounting for the social diversity and interactional context of language usage. More recently, studies using language entropy variables have shown the impact of bilingualism on cognitive and neural processes (Gullifer et al., 2018; Sulpizio et al., 2020; Li et al., 2020; Gullifer and Titone, 2021).

We routinely collect language background information about language usage and proficiency in various scenarios, such as overall daily exposure to known languages or the level of language use in communicative situations (e.g., at home, at work, in social settings). Despite their practical and theoretical importance, not many researchers have used them as covariates or predictors of behavior. One reason is that the sheer number of variables associated with bilingual experience collected in different questionnaires is daunting. Another reason for underuse is that the distribution of individual variables acquired *via* discrete replies (using the Likert scale) may not be optimal for analysis. Some of these problems can be solved with statistical manipulation of different variables to obtain an appropriate number of dependent variables. Many studies have efficiently modeled bilingual experience by using a statistical model that relates a set of observable variables to a set of latent variables, allowing to quantify bilingual experience efficiently (Anderson et al., 2018; Dash et al., 2019, 2022; Gullifer et al., 2020; Sulpizio et al., 2020). For example, Anderson et al. (2018) employed an exploratory factor analysis method to identify three variables (non-English home usage and English proficiency, non-English social use, and English use) that can describe different levels of bilingualism when all questionnaire items are included. Dash et al. (2022) used both subjective and objective measures of bilingualism and discovered three-factor structures (L2 Exposure and Proficiency–subjective, L2 Task proficiency–objective, and L2 Age of Acquisition–subjective) that had different effects on resting-state functional connectivity data. This method makes it easier for researchers to access different bilingual profiles and makes it easier to compare data from different cultural and linguistic backgrounds. These findings encourage comprehensive bilingualism tests since different features of bilingualism and the bilingual experience can have varied effects on cognitive performance. Gullifer et al. (2020) found different factor structures for language proficiency (L2 proficiency, L1 subjective proficiency, L1 objective proficiency), language entropy (Internal, external/professional, media), and language exposure (Internal, external/professional). In a recent review paper, Kremin and Byers-Heinlein (2021) proposed two methods to quantify bilingual experience using the factor mixture model and the grade of membership model. These models allow for effective accounts for bilingual language experience within categories and accommodate variations on a continuum. With the broader definition of bilingualism, there is inevitably more variation among people who are now classified as bilingual. Researchers have used factor mixture models to capture heterogeneity within groups (Clark et al., 2013; Sulpizio et al., 2020). Usually, participants are divided into groups based on the patterns of responses to the questionnaire, and each group is assigned a composite score on a continuous scale indicating their position within the group. For example, Sulpizio et al. (2020) used L2 AOA and L2 language entropy as the grouping variable and assessed the resting-state connectivity differences on a continuum of L2 proficiency. Another study by Luk and Bialystok (2013), although not assessing cognitive performance, used confirmatory factor analysis to extract two correlated factors–daily bilingual usage and English proficiency. On the other hand, a grade of membership model allows individuals to have partial membership in either of the groups based on the characteristic of the population. It is a latent structure model in which observable variables are represented as a continuous mixture of fuzzy classes; these classes account for the individual heterogeneity in bilingual groups. However, we are yet to see researchers using a grade of membership approach to the bilingual adult or aging population. Interestingly, a recent study has used a version of the graded membership approach with Spanish-speaking English learners at risk for reading difficulties attending middle school (Macdonald et al., 2022). Authors have used a combination of person-centered (using confirmatory factor analysis) and variable-centered approaches (using latent profile analysis) to characterize language skills and to identify different bilingual profiles within their study sample based on a battery of objective measures of language proficiency. However, the interrelationship of these outcome variables with cognitive performance is not directly assessed. It is crucial to note that the recommended number of participants for using the factor mixture and grade of membership models was 150-200 (Kremin and Byers-Heinlein, 2021), which was not the case in the studies mentioned above. Especially with the aging population, it is hard to reach the prescribed number. While noting the drawbacks of categorization, Grosjean (1998) proposed using one measure of bilingualism (for example, L2 proficiency) to perform regression analysis, with other related observable variables can be used as a covariate variable (i.e., L2 AOA), that may allow participants to be their controls. Given that different measures of bilingualism are interrelated to each other, one may also opt for partial correlation analysis. We expect that these scores will assist the researcher in quickly determining a proper estimation/classification of measures of bilingualism.

## Performance-based cognitive measures and their neural correlates as an index of benefits related to bilingualism

Just like “bilingualism,” “cognitive performance” also is a multifactorial reality. Beyond the intricacies of second language experience, Grosjean (1998) has stressed the necessity of taking multiple cognitive tasks into account when studying the impact of bilingualism on cognitive performance. The present debate on the role of bilingualism on cognition is suffering from oversimplified definitions for bilingualism and cognitive processes under study (Bialystok, 2021a). Usually, the impact of bilingualism is studied separately in different cognitive processes, like attention (Costa et al., 2008; Marzecová et al., 2013; Dash et al., 2019), cognitive control (Bialystok et al., 2005), working memory (Grundy and Timmer, 2017). However, cognitive processes are a multidimensional construct with interrelated thus, a single factor description of bilingualism and cognition is a reductionist approach to understanding the relationship between bilingualism and cognition (Bialystok, 2021a,b). Most of the studies discuss bilingual performance on executive function abilities using a wide variety of tasks (anti-saccade task, Stroop task, stop-signal task, letter memory task, letter-shape task, Simon task, flanker task, ANT, Wisconsin Card Sorting task, AX-CPT among others). However, performance on different cognitive tasks cannot be equated to the executive function ability and may have been influenced by other cognitive processes. In addition, other variables are known to influence executive function abilities (like education, leisure activities, socio-economic status), more so in the aging population (see Valian, 2014 for details). According to Valian (2014), lack of clarity on the definition and assessment of executive function and a lack of control over the confounding variables are the primary reasons for the discrepancies that are evident in the bilingual literature. Another example of discrepancy in the literature on the role of bilingualism in cognitive performance arises from the modality of testing the cognitive performance, i.e., by using verbal and non-verbal tasks. It is well-established that bilinguals perform poorer on verbal tasks across the lifespan (Bialystok, 2009), specifically in tasks requiring language production (Gollan et al., 2005; Sullivan et al., 2018), receptive vocabulary (Bialystok and Luk, 2012), lexical access in sentence comprehension (Shook et al., 2015) and verbal fluency (Rosselli et al., 2000). As a result, bilinguals perform worse on cognitive tasks involving verbal processing. These tasks do not correctly reflect the domain-general cognitive performance because of bilingual experiences, resulting in more evidence of sizeable cognitive advantage in non-verbal tasks. Usually, verbal fluency tasks that place more demand on the cognitive mechanism (i.e., letter fluency, Patra et al., 2020) have shown better performance in bilinguals than monolinguals, whereas, in the category fluency task, the gap between bilingual and monolingual performance is narrowed (Kormi-Nouri et al., 2012).

**Figure 1:**
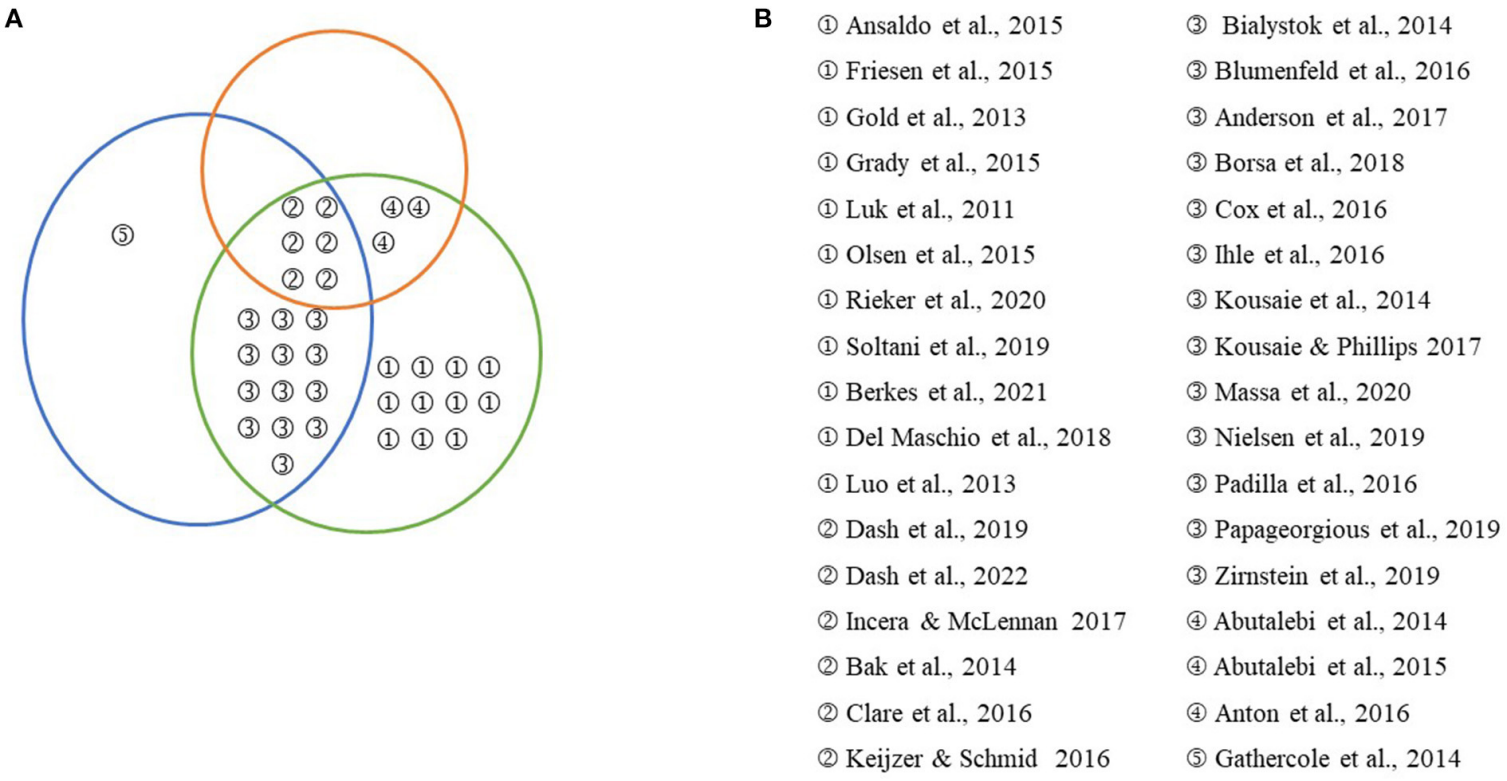
**(A)** Venn diagram to visually group bilingual research conducted with the aging population within three categories (1) the multifactorial nature of bilingualism (in Orange), (2) the multifactorial nature of cognitive performance (in Blue), and (3) the multifactorial nature of confounding variables (in Green). **(B)** Numbers refer to the references shown in the corresponding panel.

Another variability in bilingual literature is the multifactorial nature of demographic and life experiences, such as age, education, gender, social-economic status, and leisure. Some of these variables directly influence cognitive performance, whereas others relate to the bilingual experience. On average age-related cognitive decline begins in the Middle Ages (50–60 years old) and accelerate with increasing age (Ghisletta et al., 2019). Age-related changes are evident in many cognitive domains, such as memory, attention, executive function, visual perception, and linguistic abilities (Salthouse, 2004; Dash and Joanette, 2017). Furthermore, environmental influences and lifestyle choices can compensate for the magnitude of age-related changes in cognitive and structural brain alterations. Individuals with a higher premorbid IQ (Deary et al., 2004), educational (Franzmeier et al., 2017) or occupational attainment (Scarmeas and Stern, 2003), and engagement in leisure activities (Stern, 2021) may maintain cognitive ability despite age-related neural changes or neuropathology (Zahodne et al., 2015). Another demographic variable that may contribute to cognitive difference is gender. Women score higher on cognitive tasks that require verbal processing, whereas males score better on tasks that require visuospatial processing in adulthood (Hyde, 2016). A systematic study concluded that gender differences in cognitive decline are similar between 60 and 80 years. However, gender differences in cognitive decline may exist after the age of 80, albeit the directions of the relationships discovered were occasionally conflicting (Ferreira et al., 2014). Not many studies in the bilingual literature account for gender differences; studies in Figure 1 show that only 5 out 35 studies have used gender as a confounding variable. In a series of studies, Hilchey and Klein (2011) noted the significant variations in socio-cultural backgrounds of bilinguals vs. monolinguals and caution that there may be many other “hidden factors” that lead to performance discrepancies while comparing monolingual and bilingual participants.

To assess the relationship between bilingualism and cognition in older adults, we reviewed studies conducted in the past decade focusing on the methodological approaches discussed above. Figure 1 illustrates different studies that have highlighted (1) the multifactorial nature of bilingualism, (2) the multifactorial nature of cognitive performance, and (3) the multifactorial nature of confounding variables. Only a few studies have tried to assess all three aspects of multifactoriality (Keijzer and Schmid, 2016; Incera and McLennan, 2017; Dash et al., 2019, 2022). The multifactorial nature of bilingualism is established by using the measure of bilingualism on a continuum (Incera and McLennan, 2017; Dash et al., 2019, 2022) or by including subjective and multiple objective measures of bilingualism (Abutalebi et al., 2015; Keijzer and Schmid, 2016; Anderson et al., 2018) or my correlation L2 proficiency differences within the bilingual group with cognitive performance (Abutalebi et al., 2015; Antón et al., 2016; Clare et al., 2016). Dash et al. (2019, 2022) created the continuum of bilingualism by using four objective measures of language proficiency and self-reported information using LEAP-Q; and assessed the impact of bilingualism using factor scores on cognitive and neural processes. Incera and McLennan (2017) assessed participants varying in their level of L2 language usage and exposure (i.e., from completely monolingual to balanced bilinguals). It is worth acknowledging that the continuum approach is also used to study bilingualism in the younger population (DeLuca et al., 2019; Sulpizio et al., 2020). Studies using objective measures of bilingualism and self-reported information are another way to give weightage to the multifactorial nature of bilingualism (Abutalebi et al., 2015; Keijzer and Schmid, 2016; Anderson et al., 2018). Abutalebi et al. (2015) and Keijzer and Schmid (2016) used multiple measures of bilingualism to evaluate the impact of language competence on the cognitive performance of their bilingual groups (Abutalebi et al., 2015; Keijzer and Schmid, 2016). Also, Bak et al. (2014) reported differences in cognitive performance between the groups categorized based on AOA (Early vs. Late), language usage (active vs. passive) & the number of languages (2 vs. multi). However, there is a possibility of overlapping participants in different groups, and there can be substantial interaction between these categorizations.

The multifactorial nature of cognitive performance is assessed using multiple cognitive tasks to understand the impact of bilingualism. It was interesting that not all cognitive processes are impacted by bilingualism. Moreover, different aspects of bilingualism may impact different subcomponents of cognition. For example., Dash et al. (2022) reported that the objective measures of L2 proficiency, in contrast to self-reported information, as a measure of bilingualism, have a more significant potential to tap into the role of bilingualism in attentional processes. Similarly, Kousaie and Phillips (2017) reported an advantage in cognitive performance only in the Stroop task and not in the flanker and Simon task. Although Kousaie and Phillips (2017) found electrophysiological differences in task performance between groups, there was a lack of convergent validity in electrophysiological markers between tasks, suggesting that these tasks might assess different underlying mechanisms. The multifactorial nature of confounding variables is often assessed by accounting for the common demographic variables like age, education, and performance on the neuropsychological test, where groups are matched on these variables. More recently, studies are controlling for cognitive reserve variables and looking at the impact of age and bilingualism on cognitive performance (Incera and McLennan, 2017; Dash et al., 2022). Nevertheless, there is a lack of studies that account for all three aspects of multifactoriality within a single study. Figure 1 provides an exciting point of view on how researchers have used the different aspects of multifactoriality in combination. Bilingual research in the aging population will benefit from the inclusion of the three aspects of multifactoriality in forming a theoretical framework that can account for the role of bilingualism in different cognitive processes while including cognitive reserve framework (age, education, leisure, occupation) to further look at the three-way interaction.

## Conclusion: Multifactorial approach to study bilingualism: A way forward

We agree with previous authors (Bialystok, 2021a; Marian and Hayakawa, 2021) to have a transparent definition of bilingualism and use multiple tools (de Bruin, 2019) to understand the bilingual population under study. After understanding the bilingual phenotype in a particular study, the next logical step is to see if researchers want to categorize or use the measures of bilingualism on a continuum depending on the research questions. This review enumerates multiple approaches that can effectively allow the researchers to use different measures of bilingualism. However, using a larger diverse dataset and advanced statistical methods to select bilingualism-related predictor variables is recommended. Methodological rigor is needed to define and assess bilingualism and to study the impact of bilingualism on different cognitive processes and their subprocesses. The inclusion of complementary performance-based cognitive measures contributes to understanding the role of bilingualism on individual cognitive processes, ultimately translating into identifying different markers of bilingualism that may influence cognition. In addition, measures of bilingualism (age of acquisition, language usage, and proficiency) may influence language representation in the brain differently and may thus influence different aspects of cognition. Different measures of bilingualism allow for a refined perspective on the impact of bilingualism on cognition, contrarily to the conflicting results obtained with past approaches. The multifactorial continuum approach to studying bilingualism allows an in-depth look at how bilingualism may contribute to cognitive and neural advantages. Finally, growing interest in the idea of bilingualism as a proxy of cognitive reserve (Bialystok, 2021b) needs to be carefully assessed by acknowledging the interaction of bilingual experience with other life experiences like education, occupation, leisure, and socio-economic status. Future researchers should assess the interaction between bilingualism and other cognitive reserve variables on cognitive performances rather than merely controlling them in studies.

Although the current paper aims to encourage researchers to consider the multifactorial approach in studying bilingualism, we have focused predominantly on the external factors (i.e., environmental factors) related to the bilingual experience. However, previous studies have effectively addressed the impact of organism internal factors like genetics on the level of bilingualism (Vaughn and Hernandez, 2018). Similarly, studies have shown that inter-individual differences in cognitive performance (Friedman et al., 2008; Kanai and Rees, 2011; Parasuraman and Jiang, 2012) and cognitive/neural reserve (Stern, 2017; Pettigrew and Soldan, 2019; Stern et al., 2020) are influenced by biological/genetic factors. The genetic factors may interact with environmental factors (L2 usage and exposure, SES, occupation, education) to produce variations in cognitive functions like memory, attention, and language. It is beyond the scope of the current review to discuss the multifactorial nature of organism internal factors and the current review has focused on the multifactorial nature of external/environmental factors. We hope that the multifactorial nature of bilingualism, cognition, and confounding variables delineated in this review will provide a framework for researchers to create a working model of the impact of bilingualism on cognition.

## Data Availability Statement

The original contributions presented in the study are included in the article/supplementary material, further inquiries can be directed to the corresponding author/s.

## Author contributions

TD drafted the initial article. TD, AA, and YJ participated in the manuscript preparation. All authors read the final manuscript and approved it for publication.

## Funding

This work was supported by the Canadian Institutes of Health Research grant (CIHR: MOP-93542). TD was supported by the postdoctoral fellowship from FRSQ.

## Conflict of interest

The authors declare that the research was conducted in the absence of any commercial or financial relationships that could be construed as a potential conflict of interest.

## Publisher's note

All claims expressed in this article are solely those of the authors and do not necessarily represent those of their affiliated organizations, or those of the publisher, the editors and the reviewers. Any product that may be evaluated in this article, or claim that may be made by its manufacturer, is not guaranteed or endorsed by the publisher.
